# Ultra‐long‐acting recombinant insulin for the treatment of diabetes mellitus in dogs

**DOI:** 10.1111/jvim.16449

**Published:** 2022-05-27

**Authors:** Sean E. Hulsebosch, Jully Pires, Michael J. Bannasch, Thomas Lancaster, Andrea Delpero, Ramya Ragupathy, Sylaja Murikipudi, Todd Zion, Chen Gilor

**Affiliations:** ^1^ Department of Veterinary Medicine and Epidemiology University of California Davis California USA; ^2^ Veterinary Medical Teaching Hospital University of California Davis California USA; ^3^ Akston Biosciences Beverly Massachusetts USA; ^4^ Department of Small Animal Clinical Sciences University of Florida, College of Veterinary Medicine Gainesville Florida USA

**Keywords:** adherence, compliance, continuous glucose monitoring, FcRn, IgG, once‐weekly insulin

## Abstract

**Background:**

For the treatment of diabetes mellitus (DM) in dogs, novel insulins with decreased injection frequency while maintaining safety and efficacy are desirable. Insulin fused with immunoglobulin‐fragment‐crystallizable (Fc) has an ultra‐long plasma half‐life because it recycles through cells, protected from proteolysis.

**Hypothesis:**

Glycemic control can be achieved in diabetic dogs with a recombinant fusion protein of a synthetic insulin and canine Fc (AKS‐218d) administered subcutaneously once‐weekly.

**Animals:**

Five client‐owned dogs with naturally occurring DM.

**Methods:**

Prospective clinical trial in dogs with DM that were recruited from the UC Davis Veterinary Teaching Hospital and local veterinary clinics. Dogs previously controlled using intermediate‐acting insulin q12h were transitioned to once‐weekly injections of a preliminary construct identified as AKS‐218d. The dose of AKS‐218d was titrated weekly for 8 weeks based on clinical response and continuous interstitial glucose monitoring. Clinical signs, body weight, serum fructosamine concentrations, and mean interstitial glucose concentrations (IG) over the preceding week were compared between baseline (before AKS‐218d) and during the last week of treatment. Data were compared using nonparametric paired tests.

**Results:**

Once‐weekly AKS‐218d, compared to baseline twice‐daily insulin therapy, resulted in no significant changes in clinical signs, median (range) body weight (+0.4 kg [−0.5‐1.1]; *P* = .6), fructosamine concentration (−75 mmol/L [−215 to +126]; *P* = .4), or mean IG (+81 mg/dL [−282 to +144]; *P* = .8). No adverse reactions were reported.

**Conclusion:**

Control of clinical signs, body weight, and maintenance of glycemia was achieved with this once‐weekly novel insulin construct in 4 of 5 dogs.

**Abbreviations**
ADA: anti‐drug antibodies
BG: blood glucose concentration
CGM: continuous glucose monitoring system
CV: coefficient of variation
DM: diabetes mellitus
FcRn: neonatal Fc receptor
FGMS: flash glucose monitoring system
GVP: glucose variability percentage
IG: interstitial glucose concentration
SD: standard deviation

## 1 INTRODUCTION

Diabetes mellitus (DM) is a common endocrinopathy in dogs, with an estimated prevalence of 0.3%‐0.6%.^1^, ^2^ Dogs with DM are invariably insulin‐dependent.^3^ Treatment of DM in dogs relies upon chronic administration of exogenous subcutaneous (SC) insulin. For many dog owners, the need to administer twice‐daily injections adversely affects their quality of life and the perceived well‐being of their pet.^4^ Owners often cite insulin‐related issues as a major cause of anxiety, including worrying about hypoglycemic events and inability to have the dog cared for by others.^4^ Faced with the challenges of daily insulin injections, approximately 33% of dog owners elect to euthanize their dog within 1 day of diagnosis.^5^

Insulin is a 51‐amino acid peptide that tends to form hexamers, especially in the presence of zinc. After injection into the SC tissue, zinc diffuses out of the SC depot, and the hexamers break down into dimers and monomers that then diffuse into the vasculature.^6^ To extend the duration of action, traditional insulin formulations typically rely on manipulation of the rate of hexamer dissociation in the SC depot to slow insulin absorption into the blood.^6^ Furthermore, decreasing the affinity of insulin to its receptor also prolongs duration of action.^7^ With manipulation of absorption rate and receptor affinity, currently available insulin formulations have time‐action profiles suitable for use as once‐ or twice‐daily SC injections.^8^

We present data on a novel ultra‐long‐acting insulin construct (AKS‐218d) intended for once‐weekly administration in diabetic dogs. The active molecule in this formulation is a fusion protein of synthetic insulin and the canine fragment crystallizable (Fc) region of immunoglobulins. This fusion protein is designed to be nonimmunogenic is a ligand to the insulin receptor, but also binds to the host neonatal Fc receptor (FcRn). Binding to the FcRn leads to recycling of the insulin fusion molecule intracellularly, which extends its half‐life compared to native insulin.^9^ Preliminary data from healthy laboratory dogs (US patent US10961294) suggest that the half‐life of this molecule would allow for once‐weekly administration with prolonged glucose‐lowering effect. We hypothesized that in dogs with naturally occurring DM, this Fc‐insulin fusion protein would control clinical signs, body weight, and blood glucose concentrations (BG) with once‐weekly injections. This was a dose‐escalation study, with the aim of assessing the ability of once‐weekly AKS‐218d injections to maintain or improve glycemic control in diabetic dogs previously treated using conventional twice‐daily insulin administration.

## 2 MATERIALS AND METHODS

**FIGURE 1:**
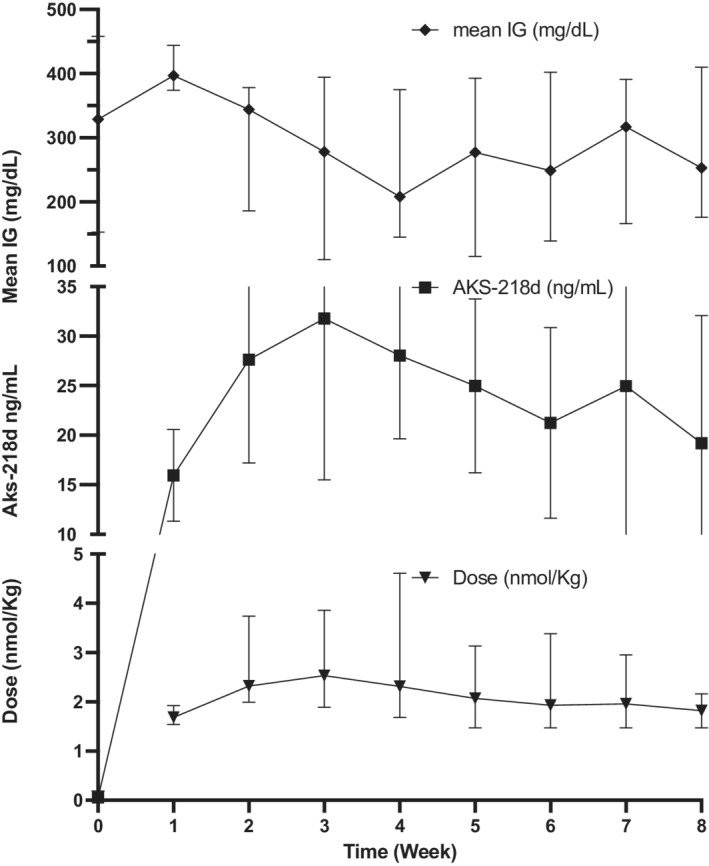
Median and interquartile ranges of AKS‐218d dose, serum AKS‐218d concentrations, and interstitial glucose (IG, mean over the preceding week) in 5 dogs treated with AKS‐218d once‐weekly. All dogs were treated with an intermediate‐acting insulin on week 0 and received the first dose of study insulin (AKS‐218d) on week 1. For each week, AKS‐218d concentrations represent the value that was obtained in the visit following the dose that correspond to the same week number. Similarly, week IG mean represent the mean of the week that followed that same dose

**FIGURE 2:**
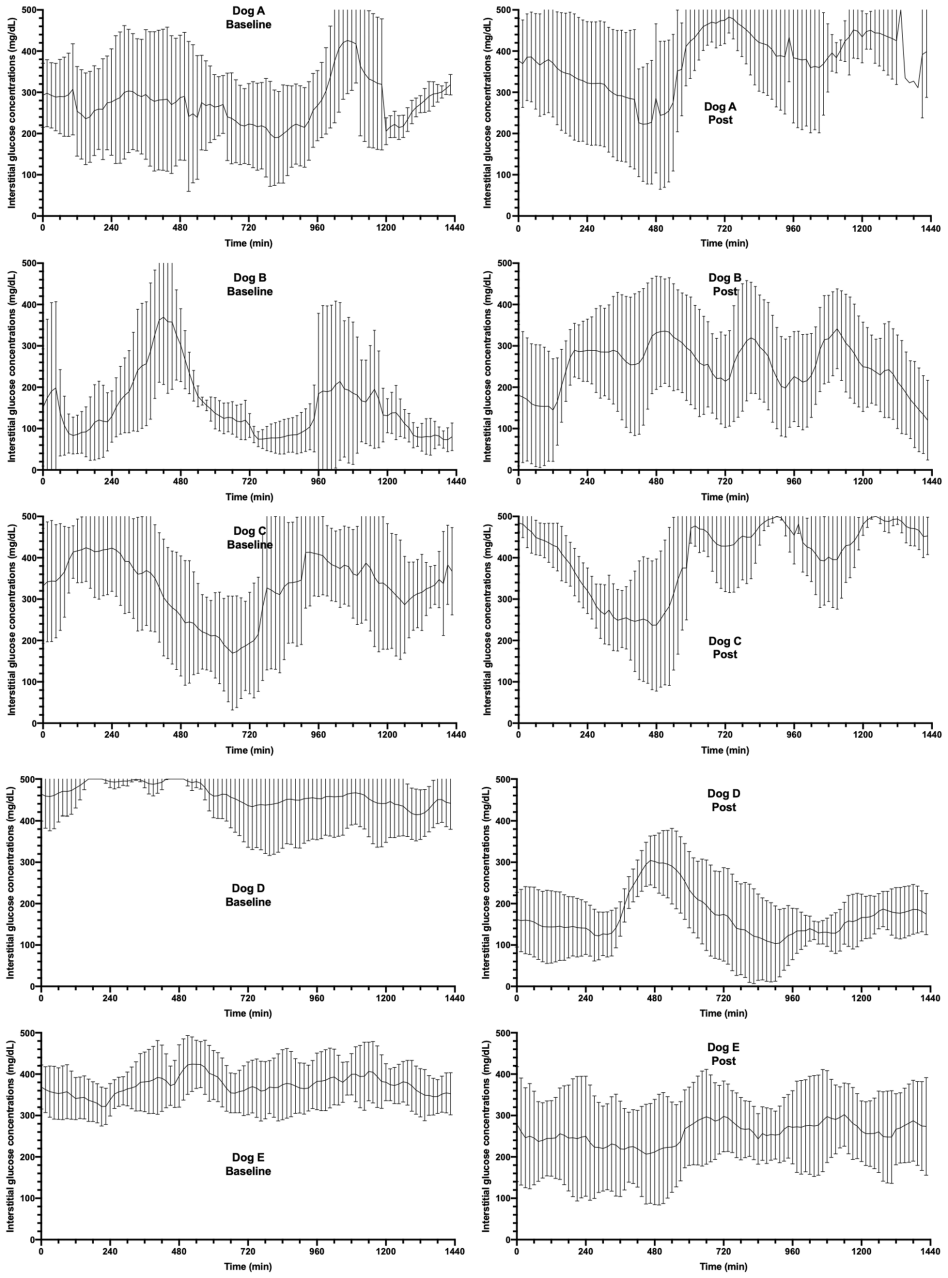
Mean interstitial glucose concentrations in 5 dogs treated with twice daily prestudy insulin (left panels: baseline) and after 7 doses (Dogs A and E) or 8 doses (Dogs B‐D) of once‐weekly AKS‐218d insulin (right panels: "Post". i.e.,final week). Each time point represents the 15‐minute mean IG over 6 days, with the line representing the mean IG and the error bars representing the SD. The X axis represents 24 hours in total

Dogs were recruited to this study from the University of California, Davis Veterinary Medical Teaching Hospital and local veterinary clinics. Dogs of any age between 3 and 30 kg with no recent changes in body weight (defined as <5% in the past 3 months) were included if they were diagnosed with naturally occurring DM (in accordance with the Project Agreeing Language in Veterinary Endocrinology [ALIVE] definition for DM diagnosis).^10^ Dogs were required to have been treated with any insulin formulation (≤1.5 U/kg per injection) for at least 2 months before enrollment. Dogs were required to have moderate to good glycemic control as defined by mild clinical signs of DM (subjectively reported by dog owners) and stable serum fructosamine concentrations (defined as 2 serum fructosamine concentrations measured at least 3 weeks apart that were within 100 mmol/L of each other). After inclusion, the degree of glycemic control on prestudy insulin was also assessed by measuring interstitial glucose concentration (IG) continuously (see below) during the first week (Figures 1 and 2). Additionally, dogs must have been fed a consistent diet throughout the study period. Exclusion criteria included any concurrent illness that might preclude following the dog >6 months and that might affect insulin requirements, poorly controlled hypothyroidism, positive urine culture, inability to tolerate the flash glucose monitor (see below) and with a history of diabetic ketoacidosis within the past 2 months, or with a history of diabetic ketoacidosis within the past 2 months.

Upon inclusion in the study, informed consent was obtained from all owners. The study protocol was approved by [masked for review]. On Visit 1, samples were collected for hematology, serum biochemistry, serum fructosamine concentration, pancreatic lipase immunoreactivity (PLI), serum trypsin‐like immunoreactivity (TLI), urinalysis (by cystocentesis), aerobic bacterial urine culture, and urine protein: creatinine ratio. All sample analyses were performed at [masked for review] clinical pathology laboratory except for PLI and TLI which were performed at the Texas A&M University Veterinary Gastrointestinal Laboratory.

A flash glucose monitoring system (FGMS; FreeStyle Libre, Abbott Laboratories, Illinois) provides clinically accurate estimates of glucose in diabetic dogs and therefore was used throughout the study.^11^ A FGMS sensor was applied to the skin on the dorsolateral lumbar area as previously described.^9^ This FGMS measures IG once per minute and records 15‐minute IG averages for up to 14 days. Minute‐by‐minute measurements can be obtained by scanning the sensor more frequently. After this initial screening visit, dogs were discharged for 10‐14 days to continue receiving their prestudy insulin twice daily and to ensure compliance with the FGMS.

On visit number 2 (day 0), thoracic radiography and abdominal sonography were performed. If no exclusion criteria were met, a new FGMS sensor was applied and the dog received the first injection of AKS‐218d at approximately 1.5 nmol/kg SC. This dose was chosen based on preliminary data from healthy purpose‐bred dogs with the intention of approximating a 0.3 U/kg/day dose of conventional insulin. Based on preliminary data from healthy purpose‐bred dogs, it was anticipated that hypoglycemia would be unlikely at this dose, that escalation of the AKS‐218d dose over the succeeding weeks would be required to maintain glycemic control, and that maximal AKS‐218d effects were expected to peak within the first 24‐36 hours after injection. Because the potency of AKS‐218d was not known in dogs with naturally occurring DM, and in order to minimize the risk of hypoglycemia, dogs were hospitalized for monitoring and hourly FGMS scans were conducted for 48 hours after the first injection. If no hypoglycemia was observed, dogs were discharged from the hospital after 48 hours of observation; if hypoglycemia leading to clinical signs was observed, IV dextrose supplementation would be administered at the discretion of the study investigators. At home, the need for any additional glycemic control between weekly AKS‐218d doses was assessed by the study investigators in conjunction with the owner. Supplemental insulin was not administered by the owner unless instructed otherwise by the study investigators; when deemed necessary, based on clinical signs including excessive polyuria, polydipsia, and polyphagia in conjunction with FGMS data indicating persistent hyperglycemia, owners were instructed to also administer prestudy insulin at half the prestudy dose to address any hyperglycemic events.

After discharge from the hospital, dogs were re‐evaluated weekly via a full physical examination including body weight, serum samples were collected for drug concentration and anti‐drug antibodies (ADA), the FGMS sensor was replaced, and AKS‐218d was administered SC. The dose of AKS‐218d was adjusted weekly as necessary based on the past week's IG concentrations, body weight, and clinical response (serum drug concentration data were not available to the study investigators during the study). In total, dogs were treated with AKS‐218d 8 times (ie, for 56 days). During the 5th visit (end of week 4) and the final visit (1 week after the last injection), samples were collected for hematology, serum biochemistry, serum fructosamine concentration, urinalysis and urine protein: creatinine ratio via cystocentesis to survey for potential adverse drug effects.

### 2.1 Measurement of serum concentrations of AKS‐218D and anti‐drug antibodies

Serum drug concentrations of AKS‐218d and ADA were measured via sandwich ELISAs specifically developed by Akston Biosciences (Beverly, Massachusetts) for the purpose of this study. To measure concentrations of AKS‐218d, microtiter plates were coated with purified anti‐insulin antibodies to capture the AKS‐218d therapeutic molecules in the serum samples. The captured serum AKS‐218d then was quantitated using a goat anti‐dog IgG‐Fc‐ horseradish peroxidase (HRP) detection antibody followed by a tetramethylbenzidine substrate system. The tetramethylbenzidine substrate changes color when it reacts with the HRP that is conjugated to the detection antibody. The enzyme substrate reaction was stopped by the addition of a stop reagent (1% H_2_SO_4_) and the color intensity was measured in a microplate reader at 450 nm.

To measure ADA, microtiter plates were coated with purified insulin. For calculating the ADA in canine IgG units, plates were also coated with 1 : 2 serial dilutions of canine IgG (Jackson Immunoresearch Laboratories, West Grove, Pennsylvania) at concentrations descending from 300 to 4.69 ng/mL to create a 7‐point pseudo‐standard curve. The captured antibodies then were quantitated using a goat anti‐feline IgG F(ab′)2‐HRP detection antibody followed by a tetramethylbenzidine substrate system. The reaction was stopped by the addition of a stop reagent (1% H_2_SO_4_) and the color intensity was measured in a microplate reader at 450 nm.

### 2.2 Statistical analysis

Statistical analysis was performed on GraphPad Prism, version 9.1 (GraphPad Software Inc, La Jolla, California). Descriptive statistics of all variables were calculated as median (range) and all comparisons were performed using nonparametric tests because of the small number of dogs in the study. Wilcoxon matched‐pairs signed‐rank tests were used for comparisons between median values of the dogs at baseline (week 0, immediately before first AKS‐218d injection) and the last week of treatment after the last AKS‐218d injection. All statistical tests were performed as 2‐tailed tests and a *P*‐value ≤.05 was considered significant. Raw IG data were extracted from the Libre CSV files for each week of the study from baseline to the week following the last AKS‐218d injection. For each week, IG data were included from midnight after sensor placement until midnight of the night before AKS‐218d injection (for a total of 6 full days, excluding the 24 hours surrounding the weekly hospital visit to minimize the effect of stress‐induced hyperglycemia). These data were used to calculate mean IG for the week as well as standard deviation (SD), coefficient of variation (CV) between days of the week, and glucose variability percentage (GVP), which was calculated as previously described.^12^ In brief, the GVP method calculates the length of IG line from continuous data by using a trigonometric analysis of the data and is used as a superior indicator of intraday BG variability.^12^ It is a more comprehensive measure of variability compared to CV and SD because it captures fluctuations in both amplitude and frequency. Data were graphed to represent measurements as obtained on the day of each visit with week number corresponding to the number of AKS‐218d injection (day 0). As such, the week mean IG and GVP of week 0 correspond to the week before visit 2 (representing treatment with standard insulin [baseline]), week mean IG and GVP of week 1 representing treatment with the first dose of AKS‐218d (days 0‐7), and week 8 mean IG and GVP representing treatment with the eighth dose of AKS‐218d (days 49‐56).

## 3 RESULTS

Six dogs were enrolled into the study. One dog, an 8‐year‐old male castrated Miniature Schnauzer with a prior history of recurrent acute pancreatitis, was excluded after the 5th injection of AKS‐218d when the dog developed severe acute pancreatitis requiring prolonged hospitalization.

Of the 5 dogs that completed the study, 1 was a spayed female and 4 were neutered males. There were 2 mixed breed dogs, 2 Yorkshire terrier mix dogs, and 1 Border Terrier dog. The median age (range) was 10 (9‐12) years. Median body weight was 9 (2.9‐25.3) kg. Median body condition score was 5 of 9 (4‐8 of 9). Dogs were fed a variety of commercial dry and canned dog foods; no diet change was made throughout the study period. All enrolled dogs were confirmed healthy enough to participate in the study as there were no clinically relevant comorbidities detected by CBC, serum biochemistry, serum PLI and TLI, urinalysis, urine culture, urine protein: creatinine ratio, abdominal sonography, or thoracic radiography.

Dogs were diagnosed with spontaneous DM a median of 7 (6‐22) months before enrollment. Prior to enrollment, 4 dogs were managed with porcine Lente insulin (Vetsulin, Merck Animal Health) at dosages of 0.89, 0.95, 2.00, and 2.76 U/kg/day, respectively, and 1 dog (Dog E) was managed with human NPH insulin (Humulin N, Eli Lilly) at a dosage of 1.59 U/kg/day. All dogs received insulin q12h. All 5 dogs were considered clinically controlled based on reports of mild clinical signs at home, stable body weight, and stable serum fructosamine concentration. At baseline, the median (range) serum fructosamine concentration was 551 (325‐709) μmol/L and the week mean IG at baseline was 329 (153‐458) mg/dL. Low IG (<40 mg/dL), consistent with hypoglycemia, was documented at baseline in the dog that received the highest dose of porcine Lente and had the lowest fructosamine concentrations (dog B).

The median starting AKS‐218d dosage was 1.68 (1.54‐1.92) nmol/kg (0.11 [0.1‐0.12] mg/kg). The AKS‐218d dose was increased in all dogs for the 2nd injection (by 25%‐95%), but was subsequently decreased in 3 of 5 dogs for the 3rd injection (by 2.5%‐28%) based on IG. One dog had a dose increase for the 4th injection, but the dose was then decreased or unchanged in all 5 dogs until the end of the study. The median final dosage of AKS‐218d was 1.88 (1.47‐2.95) nmol/kg (0.12 [0.1‐0.19] mg/kg). The final dog enrolled (Dog E) received only 7 weekly AKS‐218d injections because of supply interruptions due to Covid‐19; therefore, this dog's study exit was week 7. In Dog A, supplemental porcine Lente was administered at half the prestudy dose during the last 2 days of week 1 and during week 8 because of polyuria and polydipsia and persistent hyperglycemia. Because of the combined treatment with AKS‐218d and the supplemental administration of porcine Lente during week 8, week 8 was excluded from glucose data comparisons (including week IG mean and GVP) and instead week 7 was considered study exit for this dog. No other dog required supplemental insulin.

**FIGURE 3:**
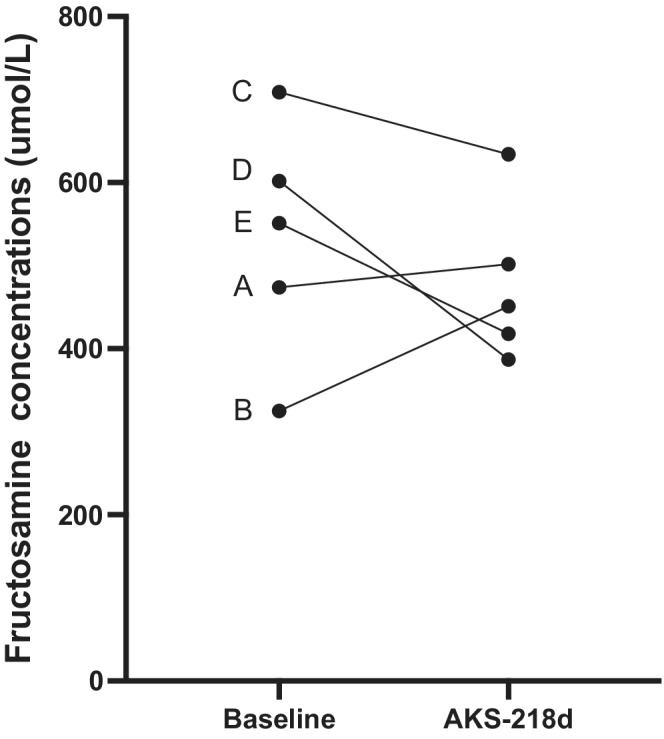
Serum fructosamine concentrations of 5 diabetic dogs (A‐E) at baseline (treated with prestudy insulin twice daily) and after treatment with AKS‐218d once‐weekly for 7‐8 weeks

**FIGURE 4:**
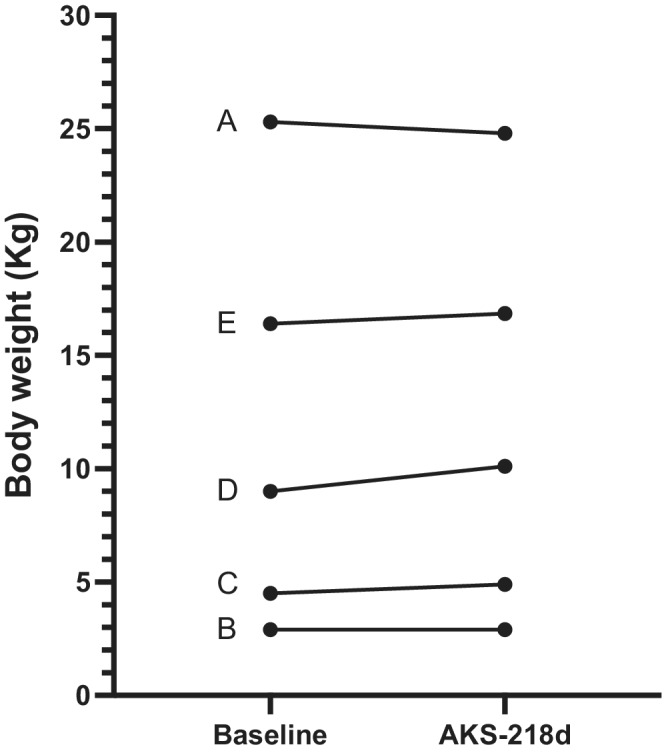
Body weight of 5 diabetic dogs (A‐E) at baseline (treated with prestudy insulin twice daily) and after treatment with AKS‐218d once‐weekly for 7‐8 weeks

At study exit (ie, week 7 for Dogs A and E, and week 8 for Dogs B, C, D), the median of week IG mean of 261 (176‐410) mg/dL was similar to baseline (*P* = .8; Figures 1 and 2) and the median serum fructosamine concentration of 451 (387‐634) μmol/L was unchanged compared to baseline (*P* = .43; Figure 3). Additionally, owners reported good control of clinical signs and body weight (10.1 [2.9‐24.8] kg) was unchanged compared to baseline (*P* = .6; Figure 4). Glucose variability was similar between AKS‐218d administration compared to baseline prestudy insulin (GVP = 204% [134‐304] vs. 210% [161‐333]; *P* = .2; Figures 1 and 2) and there was no change in CV or SD between baseline and at study exit (data not shown).

There was a moderate negative correlation between AKS‐218d serum concentration and week IG mean (*r* = −0.69 [95%CI = −0.83 to −0.47], *P* < .0001). When assessing the entirety of the data, serum AKS‐218d concentrations of approximately 20 ng/mL corresponded to good glycemic control (as represented by absence of clinical signs and a week IG mean <250 mg/dL). These concentrations were achieved in all 5 dogs a week after the second injection, with peak serum drug concentrations achieved on week 3 (Figure 1). At the end of the study, the median AKS‐218d dose was similar to week 1. Drug concentrations were maintained above 20 ng/mL in all dogs for most of the study, except for Dog A which required supplemental porcine Lente at week 8 (dog A). In this dog, the formation of ADA led to a decrease in AKS‐218d serum concentrations to 7.6 ng/mL on visit 6 and then to zero on visits 7 and 8, with a corresponding increase in mean IG; no other dog developed ADA. Serum concentrations of AKS‐218d were not available to study investigators during the clinical trial.

Interstitial glucose concentrations ≤70 mg/dL were documented in only 4.2% of all IG readings. IG concentrations ≤40 mg/dL were documented in only 0.5% of all IG readings. There was no difference in the frequency of low IG concentrations (either ≤40 or ≤70 mg/dL) between baseline (on prestudy insulin) and at study exit (*P* = .99, *P* = .75, respectively). Clinical hypoglycemia was not reported in any of the dogs; however, 1 client reported that their dog had possible listlessness 1 day during week 6 (Dog D). The event was not confirmed with a veterinary examination and the dog did not have any additional reports while continuing on study. During that week, 3.5% of all IG concentrations in that dog were ≤40 mg/dL. No adverse events were reported systemically or locally at the injection site throughout the study period, as assessed by weekly physical examination and blood and urine testing at the end of the study.

After the study ended, AKS‐218d was continued at the owners' request in the first 3 dogs to complete the trial. Dog A became refractory to treatment in the last 2 weeks of the study (requiring supplementation with twice daily insulin during week 8) was switched back to his prestudy insulin when the study ended. Two weeks later, AKS‐218d was restarted in this dog, but no clinical response was observed over 3 weeks of treatment and the dog was again switched back to twice‐daily porcine Lente insulin. Dog B remained clinically controlled for 9 months until Covid‐19 production interruptions made additional AKS‐218d unavailable. Dog C remained clinically controlled with the addition of intermittent mealtime bolus insulin (porcine Lente) for 5 months until she was euthanized after being hospitalized with acute pancreatitis at the referring veterinarian's office. Dogs D and E were switched back to their respective prestudy insulins upon completion of the clinical trial.

## 4 DISCUSSION

In our study, 5 dogs with naturally‐occurring DM were successfully transitioned from twice‐daily insulin injections to a novel construct of ultra‐long‐acting fusion insulin. The once‐weekly formulation was effective at controlling clinical signs of DM, body weight, and glycemia. These positive outcomes were achieved with no evidence of clinical hypoglycemia and without clinical, hematological or biochemical adverse events (except for infrequently low IG). Glycemic control was similar between the once‐weekly and twice‐daily protocols suggesting that the novel therapy will offer considerable benefits to the dog and pet owner.

In people, increasing dosing frequency of any drug is associated with decreased adherence to treatment protocol.^13^ In diabetic people, poor adherence is compounded by the pain of insulin injection.^14^, ^15^ Human patients consider insulin injections to be a serious burden with a negative impact on quality of life.^14^ Although adherence has not been studied in people giving insulin injections to dogs, it is anticipated that the problems noted in human patients will be compounded by the physical and emotional challenges of performing repeated injections in dogs. It is also unknown how the act of injection itself affects compliance and the owners' short and long‐term decisions to treat. Although not exclusively associated with these problems, it is reasonable to assume that the high euthanasia rate (30%‐40%) soon after DM diagnosis is associated with client's perceived compliance issues, especially when considering these results in a population of dogs covered by pet medical insurance.^4^, ^5^ A treatment that offers an alternative to daily injections should minimize these barriers to greater treatment success, improve quality of life for the pet and owner, and increase dog survival. In this context, it is also important to consider not just the treatment choice per se, but also how it affects monitoring intensity and cost.

Because ours was the first study to assess the efficacy of this novel ultra‐long‐acting insulin construct in diabetic dogs, we only recruited dogs that were well‐controlled, with the intention of comparing the treatment outcomes of the insulin‐Fc fusion protein to that of the standard insulin formulations. We did not include dogs that were newly diagnosed with DM because of an initial perception that glycemic control might take longer than usual to achieve. Considering the long duration of action of this construct and the potential for long‐term hypoglycemia in the event of an overdose, a conservative starting dose and slow rate of dose increase was advised. Although our data set is limited, it seems that a lag of about a week until full response led us to escalate the dose more then was necessary, leading to a peak at week 3 and a dose correction thereafter. Using the data from this study, it is expected that future dose exploration studies would generate a starting dose that both controls clinical signs sufficiently and avoids hypoglycemia in all dogs.

Anti‐drug antibody (ADA) formation is a common sequela of insulin therapy in dogs, cats, and humans, whether they are treated with heterologous or autologous insulin.^16^, ^17^, ^18^ The clinical importance of these antibodies is unclear, but they generally do not cause clinical problems.^8^, ^17^, ^18^ Here, ADA developed to AKS‐218d in 1 out of 5 dogs beginning about 6 weeks after starting treatment, which corresponded to a decrease in measured serum concentration of the study drug and led to the recurrence of clinical signs of diabetes. The development of these antibodies did not impede response to porcine insulin after the study ended. It remains to be established whether this antibody response was unique to the study drug construct or to administration of a heterologous insulin. During the development of the Fc‐insulin fusion construct used in this study, many Fc‐insulin fusion molecules containing different mutated insulin peptides were screened for manufacturability, bioactivity, and immunogenicity.^19^ For example, attributes like protein glycosylation can greatly influence uptake and proteolytic processing of antigens and has been implicated in many aspects of adaptive immune activation. Therefore, in the development of an insulin‐Fc protein construct, the combination of stability, high affinity and potency and long half‐life, combined with safety and insulin activity, have to be assessed and the optimal construct identified.

Considering the long duration of action of this novel insulin construct, it would likely function as a basal insulin, leading to post prandial hyperglycemia. A recent study in dogs has shown that when considering clinical signs and average glucose, monotherapy with a basal insulin can be as effective as an intermediate‐acting insulin that peaks after injection.^20^ This has also been our clinical experience with basal insulin monotherapy in dogs, with the minority of dogs requiring the addition of a meal‐time insulin to control clinical sings (manuscript in preparation). That said, a combination therapy of AKS‐218d with a shorter‐acting meal‐time insulin could be considered in dogs where tighter glycemic control is either medically necessary or desired by owners, with the goal of reducing blood glucose in the postprandial period and further optimizing glycemic control.

Considering the long duration of action of this type of novel insulin construct and that it most likely functions as a basal insulin, and as a precautionary measure to minimize the risk of hypoglycemia, we used an initial starting dose intended to approximate 0.3 U/kg/day (or 2.1 U/kg/week) with the goal of slowly increasing the dose throughout the 8 weeks of treatment, as necessary. Unexpectedly, the dose required to control diabetes at the end of the study (approximately 2 nmol/kg/week) was not significantly different than the starting dose. Dogs recruited to this study were clinically controlled with intermediate acting formulations at a median dose of 1.6 U/kg/day (or 11.1 U/kg/week) prior to participation in this study. Therefore, a “unit” of action of AKS‐218d would be approximately 0.18 nmol. This is in stark contrast to a unit of porcine Lente or NPH insulin that contain approximately 600 nmol of insulin. The relatively high potency of AKS‐218d compared to insulins porcine Lente and NPH might be explained by the increased residence time of the latter 2 insulins in the SC depot, exposing the insulin to degradation by tissue proteases, and effectively lowering the dose of intact insulin that ultimately diffuses into the blood.^21^ In contrast to other SC formulations, the prolonged duration of action of AKS‐218d does not rely on slowing absorption of the molecule from the SC tissue, but rather on intracellular circulation. The AKS‐218d is free to diffuse from the SC tissue into blood where it is distributed throughout the body. The FcRn is ubiquitously expressed in epithelia, endothelia, cells of hematopoietic origin and other cells.^22^ Upon binding to the FcRn, AKS‐218d undergoes pinocytosis and eventually exocytosis. While inside the cell, AKS‐218d is protected from proteolysis similar to other ligands of the FcRn.^22^

In most insulin formulations, retardation of absorption from the SC depot relies on the formation of insulin hexamers, a process that is a function of insulin concentration and the precise ratio of insulin to other molecules (such as zinc, protamine, or m‐cresol) and local tissue pH.^6^ This markedly limits the ability to vary the concentrations of insulin formulations and is the reason why dilute formulations have failed to maintain prolonged duration of action in the past.^23^ Because the prolonged duration of action of AKS‐218d does not rely on slowing absorption of insulin from the SC tissue, it can be formulated in any concentration, making it exceedingly convenient for use in small dogs.

An insulin that leads to lesser between‐day variability likely would require simpler monitoring protocols compared to formulations that are associated with more variability. One of the major factors contributing to between‐day variability of insulin dosage is erratic absorption of insulin crystals that vary in size and shape.^24^, ^25^ In contrast, the novel insulin construct AKS‐218d does not precipitate in the SC tissue. That, together with the fact that AKS‐218d likely achieves steady state after a few weeks of treatment (exemplified by the fact that the AKS‐218d dose remained fairly constant throughout the last 5 weeks of the study), suggested that there would be a decreased between‐day variability in the last week of the study compared to baseline. We did not observe that positive outcome, possibly because of lack of statistical power.

Interstitial glucose concentrations were measured continuously throughout the study, but these data were used primarily to aid in dose adjustments and only secondarily as outcome measures. Neither the optimal target IG nor the optimal BG in dogs is known, such that diabetic control is defined instead as “the absence of clinical signs and hypoglycemia.”^8^ In addition, although more data are available regarding BG targets, in this study we measured IG believing that it would better reflect tissue physiologic requirements for insulin and a broader perspective on trends and not just single time points.^26^ Use of a continuous glucose monitoring system (CGM) in this study of a novel ultra‐long‐acting insulin construct was believed to be important in the context of identifying subclinical hypoglycemic events. The CGM used in our study has been validated for use in dogs, showing excellent correlation between BG and IG overall.^11^ However, like other glucometers intended for use in people, this CGM underestimates glucose concentrations in the hypoglycemic range (by about 20 mg/dL).^11^ This possibly means that the frequency of low IG events in our study may be an overestimation of the frequency of true hypoglycemic events, potentially explaining why we did not observe clinical hypoglycemia. In the future, it will be important to establish a dosing scheme and develop appropriate monitoring protocols that will optimize the use of once‐weekly insulin formulations. The current study did not detect a difference in the frequency of low IG readings between q12h insulin at baseline and once‐weekly treatment with AKS‐218d at the end of the study. It is plausible that a clinically relevant difference was not detected because of the low power of this study.

In summary, once‐weekly administration of AKS‐218d was sufficient for maintaining body weight, stable serum fructosamine concentrations, and controlling clinical signs in these 5 dogs with naturally occurring diabetes mellitus.

## OFF‐LABEL ANTIMICROBIAL DECLARATION

Authors declare no off‐label use of antimicrobials.

## CONFLICT OF INTEREST DECLARATION

The authors Thomas Lancaster, Andrea Delpero, Ramya Ragupathy, Sylaja Murikipudi, and Todd Zion are from the pharmaceutical company (Akston Biosciences) that developed the insulin construct that we studied, and funded most of the study at UC Davis. The primary (Sean Hulsebosch), secondary (Jully Pires), and senior (Chen Gilor) authors have no such affiliation nor any financial interest with the pharmaceutical company.

## INSTITUTIONAL ANIMAL CARE AND USE COMMITTEE (IACUC) OR OTHER APPROVAL DECLARATION

Study design and protocol was approved by the UC Davis IACUC, protocol # 21754.

## HUMAN ETHICS APPROVAL DECLARATION

The authors declare human ethics approval was not needed for this study.
