# Erratum for “Ultra‐long‐acting recombinant insulin for the treatment of diabetes mellitus in dogs”

**DOI:** 10.1111/jvim.16667

**Published:** 2023-02-21

First published: 27 May 2022 https://onlinelibrary.wiley.com/doi/10.1111/jvim.16449

Volume 36, Issue 4

Section 1 Introduction, 3rd paragraph, 3rd sentence, the word “and” is missing. The sentence should be as follows. “This fusion protein is designed to be nonimmunogenic and is a ligand to the insulin receptor, but also binds to the host neonatal Fc receptor (FcRn).

Section 2 Materials and Methods, 1st paragraph, last sentence, a section is redundant and should be as follows. “Exclusion criteria included any concurrent illness that might preclude following the dog >6 months and that might affect insulin requirements, poorly controlled hypothyroidism, positive urine culture, inability to tolerate the flash glucose monitor (see below), or with a history of diabetic ketoacidosis within the past 2 months.”

Section 2 Materials and Methods, 2nd paragraph, 2nd sentence, “[masked for review]” should be replaced with “University of California, Davis, Institutional Animal Care and Use Committee (IACUC),” and the last sentence “[masked for review]” should be replaced with “University of California, Davis, Veterinary Medical Teaching Hospital.”

